# Comparison of efavirenz levels in blood and hair with pharmacy refills as measures of adherence and predictors of viral suppression among people living with HIV in Nigeria

**DOI:** 10.1186/s12981-022-00462-3

**Published:** 2022-07-10

**Authors:** Jacinta N. Nwogu, Samuel O. Ngene, Chinedum P. Babalola, Adeniyi Olagunju, Andrew Owen, Saye H. Khoo, Olayinka A. Kotila, Baiba Berzins, Hideaki Okochi, Regina Tallerico, Monica Gandhi, Babafemi Taiwo

**Affiliations:** 1 Department of Pharmaceutical Chemistry, Faculty of Pharmacy, Lead City University, Ibadan, Nigeria; 2 Department of Pharmaceutical Chemistry, Faculty of Pharmacy, University of Ibadan, Ibadan, Nigeria; 3 Department of Epidemiology and Medical Statistics, Faculty of Public Health, University of Ibadan, Ibadan, Nigeria; 4 Centre for Drug Discovery Development and Production (CDDDP), Faculty of Pharmacy, University of Ibadan, Ibadan, Nigeria; 5 Department of Pharmacology and Therapeutics, University of Liverpool, Liverpool, UK; 6 Division of Infectious Diseases and Institute for Global Health, Northwestern University, Chicago, USA; 7 Division of HIV, Infectious Diseases, and Global Medicine, University of California, San Francisco, USA

**Keywords:** Hair, Adherence, Dried blood spots, Efavirenz concentrations, Viral suppression, Pharmacy refill adherence

## Abstract

**Background:**

Strategies to support adherence are constrained by the lack of tools to objectively monitor medication intake in low-resource settings. Pharmacologic measures are objective, but pharmacy refill data is more accessible and cost-efficient. This study compared short-term and long-term efavirenz (EFV) drug levels with pharmacy refill adherence data (PRA) and evaluated their ability to predict viral suppression among people living with HIV in Nigeria.

**Methods:**

Paired hair and dried blood spot (DBS) samples were obtained from 91 adults living with HIV receiving 600 mg EFV-based antiretroviral therapy (ART) and EFV concentrations were measured via validated methods using liquid-chromatography-mass-spectrometry. PRA was estimated from pharmacy records, based on the number of days a patient collected medication before or after the scheduled pick-up date. PRA was categorized into ≤ 74%, 75–94% and ≥ 95%, defined as poor, medium and high adherence, respectively. HIV viral loads closest to the hair sampling time (within 6 months) were also abstracted. Receiver Operating Characteristics (ROC) curve analyses compared the ability of adherence metrics to predict viral suppression**.**

**Results:**

Based on PRA, 81% of participants had high adherence while 11% and 8% had medium and poor adherence, respectively. The median (IQR) EFV concentrations were 6.85 ng/mg (4.56–10.93) for hair and 1495.6 ng/ml (1050.7–2365.8) for DBS. Of the three measures of adherence, hair EFV concentration had the highest Area Under Curve (AUC) to predict viral suppression. Correlations between EFV concentrations in DBS and hair with PRA were positive (r = 0.12, P = 0.27 and r = 0.21, P = 0.05, respectively) but not strong.

**Conclusions:**

EFV concentrations in hair were the strongest predictor of viral suppression and only weakly correlated with pharmacy refill adherence data in Nigeria. This study suggests that resource-limited settings may benefit from objective adherence metrics to monitor and support adherence.

## Background

The efforts to end global HIV epidemics across every region of the world have yielded commendable results. Management of HIV disease involves several components including, early diagnosis, early initiation of antiretroviral therapy (ART), and ongoing adherence to medications. ART adherence is difficult to maintain, yet good adherence to ART is critical, particularly in resource-limited settings where viral load and resistance testing may not be routinely available to monitor ART outcomes. In addition, strategies to support adherence are constrained by the lack of tools to objectively monitor medication intake in low-resource settings. Although different ART regimens have different levels of adherence “forgiveness”, even recent studies have shown that an adherence rate ≥ 95% is associated with the most favorable treatment outcomes [1, 2]. Poor adherence to ART has been associated with lower rates of virologic suppression [3–6] and inflammation [7].

There is currently no gold standard method to accurately and independently measure medication adherence since each available method has inherent limitations. Pharmacologic measures are objective but pharmacy refill data is more accessible and cost-efficient. These methods also tend to be poorly correlated, including self-report, pill counts, electronic adherence monitors (e.g., medication event monitoring system), pharmacologic monitoring (e.g., drug levels in hair, plasma, dried blood spots (DBS), urine) and pharmacy refill adherence methods. Illustratively, self-reported adherence has shown poor agreement with pharmacologic metrics in many studies [6, 8–10], and, while medication event monitoring systems correlated well with pharmacologic methods [10], an inverse relationship between pharmacy refill data and DBS drug levels has been reported [11]. The advantages and disadvantages of each method have been well documented in previous reports [12–16].

Of the methods available to measure adherence, pharmacologic metrics are the most objective for monitoring actual drug ingestion, even though they are also imperfect. One major limitation of using antiretroviral drug levels in plasma (or levels in DBS for drugs not processed intracellularly, such as efavirenz) to measure adherence is that these metrics assess short-term exposure (within few days) and are thereby subject to “white coat compliance” [15], where patients increase their adherence to medication prior to clinic visits. Consequently, high adherence may be falsely recorded from short-term metrics. Measurements of drug concentrations in hair (or in DBS for intracellularly processed medications such as tenofovir) provides evidence of adherence for longer periods, up to weeks. Given that no single metric of adherence serves as a gold standard, a combination of methods has been suggested [2, 12, 16] in order to allow for a more robust assessment. For example, a combination of plasma (or DBS for most drugs) and hair drug levels can be used to determine short-term and long-term adherence, respectively. High drug concentrations in both matrices would indicate good adherence but discordant results with low levels in hair and high levels in plasma/DBS would provide evidence for white coat compliance.

In some resource-limited settings like Nigeria, pharmacy refill data or self-report are the most commonly-used methods to estimate patients’ adherence, although no previous study has confirmed their reliability in this population. To address this gap in the literature, our study compared pharmacy refill adherence data with drug levels in hair and dried blood spots among people living with HIV on efavirenz-based regimens in Nigeria.

## Methods

### Participants’ recruitment and sample collection

A cross-sectional study was conducted in the HIV clinic in a tertiary institution in South West Nigeria. Consenting patients receiving 600 mg efavirenz-based ART for at least 2 months were randomly selected in the waiting room of the HIV clinic at each clinic day until the required sample size was reached. A sample size of 91 was estimated with the single proportion formula considering that PRA adherence was 27% from an African study [17]; this sample size provides a 5% degree of precision and 5% type 1 error in a population less than 10,000 [18]. Hair and DBS samples were collected from the ninety-one (91) adult participants who gave informed consent.

Participants’ demographics and HIV viral loads closest to the hair sampling time (within 6 months) were abstracted from medical records. Pharmacy refill adherence (PRA) was estimated as a percentage from the pharmacy records, based on the number of days a patient collected medications before or after the scheduled medication pick-up date in line with the method used in estimating adherence at the Infectious Disease Institute, College of Medicine, University of Ibadan.

PRA was categorized into ≤ 74%, 75–94% and ≥ 95% [19], defined as poor, medium and high adherence, respectively. However, based on the receiver operator curve analysis, ≤ 75% or ≤ 85% or ≤ 95 or ≤ 100% of pills as estimated by the PRA were examined as threshold levels to predict viral suppression [20]. Ethical approval (NHREC/0/01/2008a) for this study was obtained from the joint UI/UCH ethical review board, University of Ibadan, Nigeria.

### Hair and dried blood sample collections

The procedure for hair sample collection has been described elsewhere [21]. In brief, a small thatch of hair (20–30 strands) was cut with scissors as close as possible to the scalp and properly sealed as required. The hair samples were stored at room temperature until shipment to the Hair Analytical Laboratory (HAL) at the University of California San Francisco (UCSF), USA for quantification of efavirenz levels. For DBS, venous blood was collected from each participant and spotted on to Whatmann 903 protein saver cards (50 µl per spot). The procedure used has been previously reported [22].

### Analyses of Efavirenz concentrations in hair and DBS

Efavirenz was quantified in hair using previously described and validated LC–MS/MS-based methods. Details of this procedure at the Hair Analytical Laboratory at UCSF has been described elsewhere [23] and the validated methods approved by Clinical Pharmacology and Quality Assurance Program of the National Institutes of Health [24]. Analysis of efavirenz concentrations in DBS was carried out at the Liverpool Bio-analytical Facility (BAF), Department of Molecular and Clinical Pharmacology, Institute of Translational Medicine, University of Liverpool using validated liquid chromatography tandem mass spectrometry (LC–MS/MS)-based methods [25]. Details of the laboratory methods used in this study have been reported previously [22].

### Statistical analysis

The association between hair or DBS (blood) efavirenz levels (which represents a short-term metric of exposure since efavirenz is not processed intracellularly) with PRA were assessed. The relationships between PRA and age, gender, and viral load were also assessed. Statistical analysis was carried out using IBM SPSS statistics version 25. Spearman rank correlation was used to assess the relationship between PRA, pharmacologic adherence metrics (efavirenz levels in hair and DBS), and the relevant covariates such as age, gender and viral load. Chi square statistics assessed differences in adherence by gender. Normality of continuous variables was tested using Shapiro–Wilk tests, Q-Q plots and histogram plots. Mean (standard deviation) or median (interquartile range) were used to summarize continuous variables while categorical variables were summarized by frequency and percentages in a descriptive analysis.

A receiver operator characteristic (ROC) curve of the association between specificity and sensitivity was generated to evaluate the predictive value of PRA, hair EFV concentrations and DBS EFV concentrations in forecasting viral suppression. The ROC curve was constructed by plotting the false positive rate (1-specificity) against the true positive rate (sensitivity). Detectable viral load was defined as greater than 50 copies/ml or greater than 1000 copies/ml [26, 27]. The adherence measure with the greatest total area was interpreted as having the best prediction of viral load suppression. Positive predictive value (PPV) and negative predictive value were also calculated. All analyses were performed at a significance level of 0.05.

## Results

The respondents were 64 females (70.3%) and 27 males (29.7%), with a mean ± SD age of 44.5 ± 11.2 years and median (IQR) weight of 63 (64.0–73.0) Kg. The median (IQR) duration of EFV use was 45 (23.5–70.0) months. The concomitant antiretroviral agents were tenofovir disoproxil fumarate/lamivudine (TDF/3TC) (92.3%), abacavir/lamivudine (ABC/3TC) (5.5%) and TDF /emtricitabine (TDF/FTC) (2.2%). The PRA data revealed that 8%, 11% and 81% had poor, medium and high adherence, respectively. PRA was higher among females than males though not statistically significant (P = 0.472). Paired hair and dried blood spot (DBS) samples were analyzed successfully from all 91 participants.

**Fig. 1:**
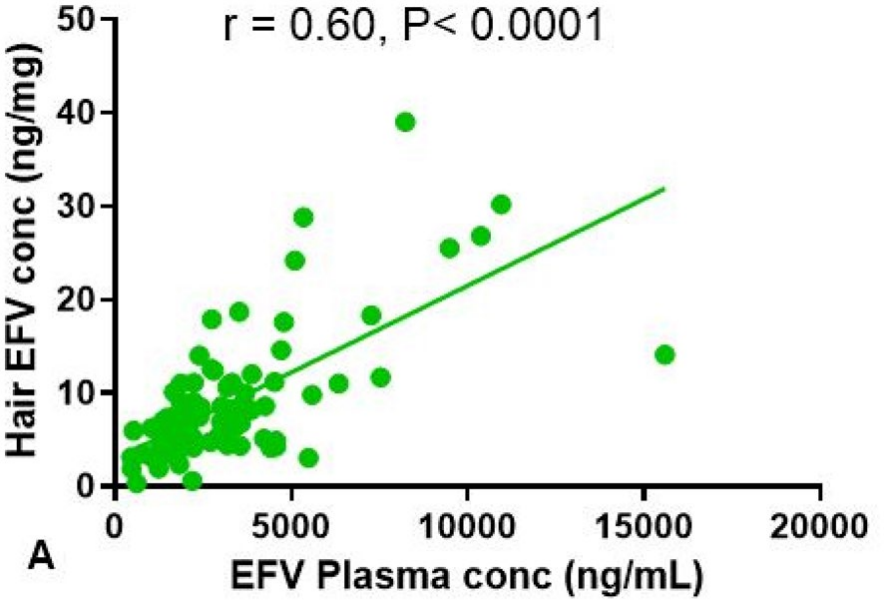
Correlation plot of hair EFV concentrations vs plasma EFV concentrations. Spearman r = 0.60 (CI 0.001425, 0.002286)

The median (IQR) EFV concentration was 6.85 ng/mg (4.56–10.93) in hair while the median EFV concentration in blood was 1495.6 ng/ml (1050.7–2365.8). There was a strong correlation between EFV levels in hair and blood (r = 0.61, P < 0.0001) as shown in Fig. 1. The correlations between EFV concentrations in blood and hair with PRA were positive (r = 0.12, P = 0.27 and r = 0.21, P = 0.05, respectively) but weaker for blood levels. Age showed a trend with PRA, where younger participants tended to have better PRA compared to older participants (r = − 0.18, P = 0.08). Higher PRA and efavirenz DBS (blood) levels were not associated with lower HIV viral loads (r = − 0.12, P = 0.34; r = 0.13, P = 0.30, respectively). Higher hair concentrations also did not show significant association with HIV viral loads (r = − 0.14, P = 0.27). There was no significant correlation between DBS and hair EFV concentration with PRA (P > 0.05). However, the median DBS and hair EFV concentrations increased with increasing level of PRA (Table 1).

**Table 1:** Summary of PRA by DBS and hair concentration

PRA category	DBS EFV concentration (ng/mL		Hair EFV concentration(ng/mg)	
	Median (IQR)	Mean rank	Median (IQR)	Mean rank
≤ 74%	1183.68 (915.28–2886.43)	41.14	4.25 (4.12–10.1)	31.50
75–94%	1525.86 (1013.14–2795.44)	48.10	6.58 (4.67–10.29)	44.75
≥ 95%	1577.62 (1053.64–229,320)	46.18	6.96 (4.76–11.05)	46.18
P value	0.33			

*PRA *pharmacy refill adherence, *IQR *median inter-quartile range

**Fig. 2:**
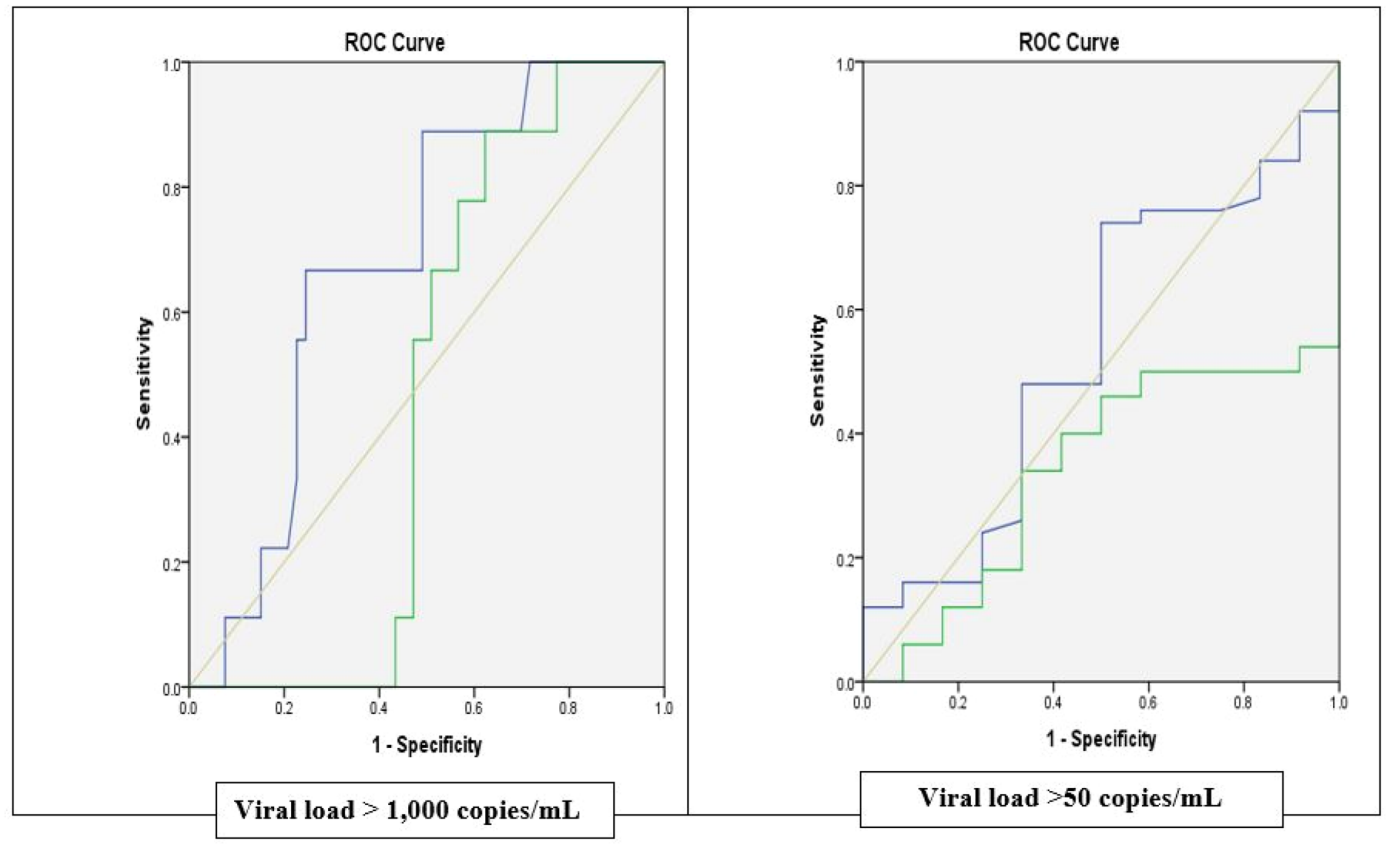
Receiver operating characteristic curve showing the relationship between adherence (DBS EFV & Hair EFV concentration) and viral load (> 50 copies/ml and > 1000 copies/ml). *Blue line *hair conc.; *Green Line *DBS conc.; *Red diagonal *reference

The area under the Receiver Operating Characteristics Curve (AUROC) for predicting detectable viral loads using DBS and hair EFV concentrations ranged from 0.34 to 0.69 and were not significantly different across the two measures of detectable viral loads (P > 0.05) (Table 2). However, hair EFV concentrations had a higher AUROC to predict viral loads than DBS EFV concentrations at viral loads > 50 copies/mL (0.54 for hair vs 0.34 for DBS) and viral loads > 1000 copies/mL (0.69 for hair vs 0.47 for DBS) (Fig. 2 and Table 2).

**Table 2:** Area under the curve for the relationship between adherence (DBS EFV & Hair EFV concentration) and viral load (> 50 copies/ml and > 1000 copies/ml)

Test results	AUROC	Std error	95% confidence interval		p value
			Upper	Lower	
Viral load > 50 copies/mL					
Hair EFV concentration	0.54	0.09	0.35	0.72	0.70
DBS EFV concentration	0.34	0.07	0.20	0.49	0.09
Viral load > 1000 copies/mL					
Hair EFV concentration	0.69	0.08	0.53	0.84	0.08
DBS EFV concentration	0.47	0.07	0.33	0.61	0.76

*AUC* Area under the receiver operating characteristics curve

Sensitivity, specificity, PPV, NPV of using PRA adherence to predict detectable viral load are presented in Table 3. At viral loads > 50 copies/mL, all four measures of PRA had high sensitivity while at viral load > 1000 copies/mL, the four levels of PRA had lower sensitivity. There was no significant difference in sensitivity, specificity, PPV and NPV at the different levels of PRA in terms of predicting viral loads (P > 0.05) (Table 3).

**Table 3:** Pharmacy Refill Adherence (PRA) prediction of detectable viral load

PRA/viral load	Sensitivity (%)	Specificity (%)	PPV (%)	NPV (%)	p value
> 50 copies/ml					
≤ 100%	70.0	14.0	27.5	50.0	0.13
≤ 95%	78.6	18.4	21.6	75.0	0.80
≤ 85%	66.7	18.3	3.9	91.7	0.52
≤ 75%	83.0	30.0	86.3	25.0	0.34
> 1000 copies/ml					
≤ 100%	15.0	86.0	33.3	68.5	0.91
≤ 95%	14.3	85.7	77.8	14.8	1.0
≤ 85%	33.3	86.7	11.1	96.3	0.33
≤ 75%	13.2	80.0	22.2	77.8	0.57

*PPV* positive predictive value; *NPV* negative predictive value; *AUC *0.565

## Discussion

Accurate assessment of antiretroviral treatment adherence is necessary to tailor adherence support. Although pharmacy refill data is often used as an objective measure of adherence in Nigeria, our study showed a poor correlation between pharmacy refill data and pharmacologic methods of measuring adherence. Pharmacologic metrics, in which drug concentrations are measured in biological matrices such as hair, DBS, plasma, and urine, provide real evidence of medication ingestion. For drugs that are not processed intracellularly (like efavirenz), plasma, urine and DBS levels provide short-term metrics of exposure whereas hair levels provide long-term information on adherence. In our study, pharmacy refill data correlated poorly with short-and-long term objective metrics of efavirenz ingestion (Table 2). Previous reports have shown similar results where pharmacy refill data showed inverse correlations with tenofovir diphosphate (TFV-DP) drug levels in DBS [11].

Multiple studies have shown hair antiretroviral concentrations to be a strong predictor of virologic outcome [19, 28–31], with a systematic review and meta-analysis of these studies verifying this predictive utility [32]. Similarly, our study (the first to be performed in Nigeria) showed that hair concentrations of ART were predictive of virologic failure, verifying their utility as a tool to objectively monitor antiretroviral medication adherence. Traditionally, adherence >  = 95% was the preferred threshold for maintaining viral suppression in people living with HIV [33, 34], but our study and others [35] suggest that lower levels (> 80%) of adherence can also be associated with relatively high rates of viral suppression. In ROC analyses in this study, the AUC for hair concentrations in predicting virologic suppression (at either ≥ 50 copies/mL and ≥ 1000 copies/mL) was higher than either DBS levels or PRA.

In terms of correlation analyses, only weak positive correlations were found between pharmacy refill adherence and EFV concentrations in blood (r = 0.12, P = 0.27) and hair (r = 0.21, P = 0.05). Among study participants, 81% had high adherence (> 95%) estimated by PRA, but this high level of adherence was not verified by objective pharmacologic measures. This may mean that some participants consistently pick up their medication but may not consume them as prescribed. These results indicate that relying on pharmacy refill data as a sole method of assessing adherence in our population is not sufficient. The scale-up of pharmacologic methods to measure and support adherence in resource-limited settings could lead to better therapeutic outcomes.

Hair concentrations of antiretrovirals have several advantages when compared to other methods of assessing adherence. Self-reported adherence is usually associated with recall bias (patients may not be able to recall all missed doses) or social desirability bias, whereby patients respond to questions to please the researcher/clinicians even when they had poor adherence. In a study among Ugandan breast-feeding women on antiretroviral therapy, hair drug concentration was the strongest predictor of viral suppression while self-reported adherence did not show significant association with virologic outcomes [29]. Other methods of assessing adherence such as pill counts, medication events monitoring systems (MEMS), or pharmacy refill data, have similar shortfalls. For instance, not all MEMS cap openings amount to medication consumption [36]; moreover, network failures can affect the accuracy and reliability of MEMS as a sole method for adherence monitoring, especially in resource constrained settings. The accuracy of pill counts is also based on the assumption that all missing pills have been consumed. Finally, hair is easy to collect, has no biohazardous precautions, can be stored at room temperature, and hair measurements can be performed in resource-constrained settings [8], providing some advantages over blood-based methods. While socio-cultural factors such as fear of rituals may limit the willingness of some Nigerians to accept hair sampling [37], acceptance is generally high [38].

The major limitation of our study is that viral load measurements were not carried out at the same time as hair and DBS sample collection. Viral loads were determined from pharmacy records within a six-month period before sample collection. Actual viral loads at the time of sample collection may have varied from the viral load obtained from records for some participants. However, the majority of the participants (51 out of 63) already achieved sustained viral suppression, with viral loads less than 50 copies/mL. Therefore, variation in viral load may be very minimal within the specified period.

## Conclusion

In this Nigeria-based study, pharmacy refill adherence data was not well correlated with objective adherence metrics (efavirenz levels in hair and blood), suggesting that pharmacy refill data may not accurately quantify ART ingestion in our population. Non-adherent patients may collect medicines but not take daily doses. Moreover, PRA and blood efavirenz levels were not significantly associated with HIV viral loads. The metric most associated with virologic suppression in receiver operating characteristic (ROC) analyses was hair ART levels. Resource-limited settings require easy-to-perform objective adherence metrics in order to monitor and support adherence.

## Data Availability

The datasets generated and/or analysed during the current study are not publicly available due to the fact that consent to make the data publicly available was not included in the informed consent process but the data are available from the corresponding author on reasonable request.
